# A new approach by optical coherence tomography for elucidating biofilm formation by emergent *Candida* species

**DOI:** 10.1371/journal.pone.0188020

**Published:** 2017-11-16

**Authors:** Melyna Chaves Leite de Andrade, Marcos Andre Soares de Oliveira, Franz de Assis Graciano dos Santos, Pamella de Brito Ximenes Vilela, Michellangelo Nunes da Silva, Danielle Patrícia Cerqueira Macêdo, Reginaldo Gonçalves de Lima Neto, Henrique Jonh Pereira Neves, Ildnay de Souza Lima Brandão, Guilherme Maranhão Chaves, Renato Evangelista de Araujo, Rejane Pereira Neves

**Affiliations:** 1 Department of Mycology, Federal University of Pernambuco, Recife, Brazil; 2 Department of Electronics and Systems, Federal University of Pernambuco, Recife, Brazil; 3 Department of Pharmaceutical Sciences, Federal University of Pernambuco, Recife, Brazil; 4 Department of Tropical Medicine, Federal University of Pernambuco, Recife, Brazil; 5 Department of Clinical and Toxicological Analysis, Federal University of Rio Grande do Norte, Natal, Brazil; University of Wisconsin Medical School, UNITED STATES

## Abstract

The majority of microorganisms present a community lifestyle, establishing biofilm ecosystems. However, little is known about its formation in emergent *Candida* species involved in catheter-related infections. Thus, various techniques may be used in the biofilm detection to elucidate structure and clinical impact. In this context, we report the ability of emergent *Candida* species (*Candida haemulonii*, *C*. *lusitaniae*, *C*. *pelliculosa*, *C*.*guilliermondii*, *C*. *famata* and *C*. *ciferrii*) on developing well structured biofilms with cell viability and architecture, using optical coherence tomography (OCT). This new approach was compared with XTT analyses and Scanning Electron Microscopy (SEM). A positive correlation between oxidative activity (XTT) and OCT results (r = 0.8752, *p* < 0.0001) was observed. SEM images demonstrated cells attachment, multilayer and morphologic characteristics of the biofilm structure. *C*. *lusitaniae* was the emergent species which revealed the highest scattering extension length and oxidative metabolism when evaluated by OCT and XTT methods, respectively. Herein, information on *C*. *ciferri* biofilm structure were presented for the first time. The OCT results are independently among *Candida* strains and no species-specific pattern was observed. Our findings strongly contribute for clinical management based on the knowledge of pathogenicity mechanisms involving emergent yeasts.

## Introduction

Several biological mechanisms involved in fungal infections are not yet fully understood. Therefore, a major concern involves the understanding and characterization of emergent *Candida* species [1]. The sudden emergence of previously uncommon and apparently harmless yeast species as agents of invasive candidiasis may be attributed to several factors such as the use of medical devices including catheter and biofilm formation [2,3]. In particular, systemic infections by emergent yeasts as *Candida ciferrii*, *C*. *famata*, *C*. *guilliermondii*, *C*. *haemulonii*, *C*. *lusitaniae*, and *C*. *pelliculosa*, had increased the mortality rate, especially when associated with severe underlying pathologies and the use of medical devices [4].

Interestingly, the majority of microorganisms survive in nature because of their community style of life as biofilm ecosystems [5]. Biofilms are structured microbial communities attached to either biotic or abiotic surface embedded in an exopolymeric matrix constituted mainly by carbohydrates, hexosamines, uronic acids, proteins and nucleic acids [6]. The biofilm architecture maintenance may be ensured by the formation of canals and columns, which allow the passage of nutrients and oxygen for the entire microbial community.

The biofilm formation is essential for yeasts protection against host defense mechanisms and commonly used antifungal drugs. Biofilm formation in implant devices is also an important medical concern, leading to clinical failure [7,8]. During the last decades, unusual yeast species have emerged as agents of invasive candidiasis and strong cause of death [9].

Classically, *C*. *albicans* develop highly structured biofilms with multiple cell types as budding yeast-form cells, pseudohyphae and true hyphae encased in an extracellular matrix. Commonly, *Candida* non-*albicans* biofilms form extracellular matrix but do not produce true hyphae [10]. Thus, its formation is an important feature for yeast virulence, and studies regarding this complex structure by emergent *Candida* species are still incipient [11, 12]. Various techniques may be used in the biofilm detection commonly SEM and metabolic activity evaluation by XTT (2,3-Bis-(2-Methoxy-4-Nitro-5-Sulfophenyl)-2H-Tetrazolium-5-Carboxanilide inner salt) [12,13]. Although rarely applied, imaging techniques may be an option in the detection of fungal biofilm. Thus, optical coherence tomography (OCT) is a well-established, low-coherence interferometric technique that performs high-resolution, ultrafast, noninvasive, and cross-sectional tomographic imaging, This optical technique evaluates interference patterns of backscattering light to build images, in depth, of biological structures as *Candida albicans* biofilm [14, 15]. In this context, the purpose of our study was to evaluate the potential use of OCT on analyzing the ability of emergent *Candida* species (*Candida haemulonii*, *C*. *lusitaniae*, *C*. *pelliculosa*, *C*. *guilliermondii*, *C*. *famata* and *C*. *ciferrii*) to develop well structured biofilms with cell viability and architecture.

## Results

### Emergent *Candida* strains

The emergent *Candida* species included in the present study were as follows: *Candida haemulonii* (3 strains), *C*. *lusitaniae* (3), *C*. *pelliculosa* (3), *C*. *guilliermondii* (4), *C*. *famata* (1) and *C*. *ciferri* (1). All yeast cultures evaluated in this study were previously isolated from critically ill patients, identified by MALDI TOF-MS and then were kept in the URM Culture Collection, Pernambuco, Brazil.

### Quantitative analyses of biofilms: Oxidative activity and optical coherence tomography (OCT)

**Fig 1:**
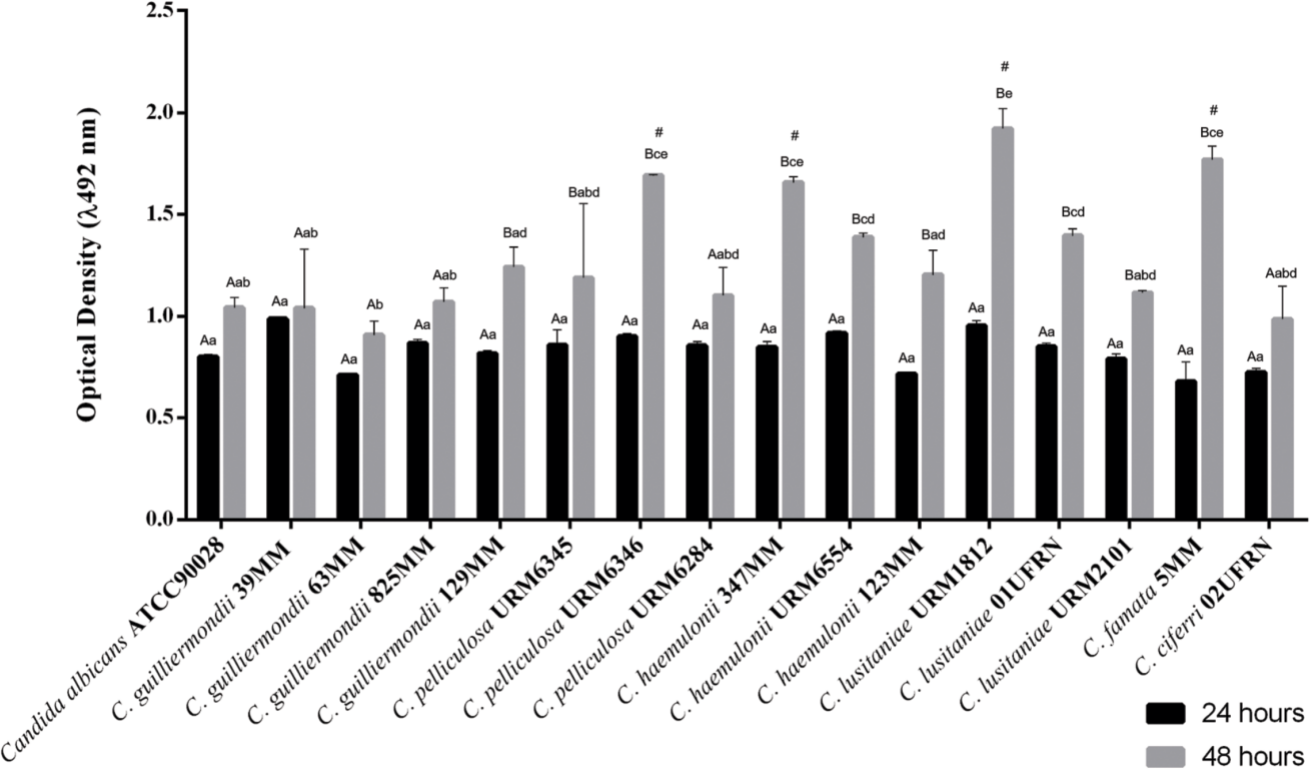
Oxidative activity biofilm for emergent *Candida* strains developed at 24 and 48 hours. Data represent the mean and standard deviation (SD) of the XTT absorbance during biofilm production in two independent experiments with at least three replicates (n≥ 6). For the analysis, Tukey's multiple comparisons test was performed for all averages obtained at the 5% level of significance. Different capital letters indicate significant difference in biofilm production in relation to time (24 and 48 hours) for a single *Candida* isolate. Different lowercase letters indicate significant difference in biofilm production among *Candida* isolates. The "#" symbol represents the isolates that have excelled in biofilm production in relation to the others, but they do not differ each other.

During the oxidative activity with the colorimetric assays based on XTT reduction, we observed that the emergent *Candida* strains used in the study were able to form an active biofilm. Quantitative XTT analyses revealed that mature stages with highest metabolic activities occurred at 48 hours of incubation (Fig 1).

Fig 1. shows the mean OD 492 nm for each strain, for biofilms formed after 24 and 48 hours of incubation. *C*. *guilliermondii* strains did not present a significant variation for biofilm formation detected by XTT activity, exhibiting a homogenous quantitative pattern. This characteristic was not verified among the other emergent strains, such as *C*. *pelliculosa*, *C*. *haemulonii* and *C*. *lusitaniae*, which presented considerable differences in optical densities caused by XTT activity values for the strains within the same species.

**Fig 2:**
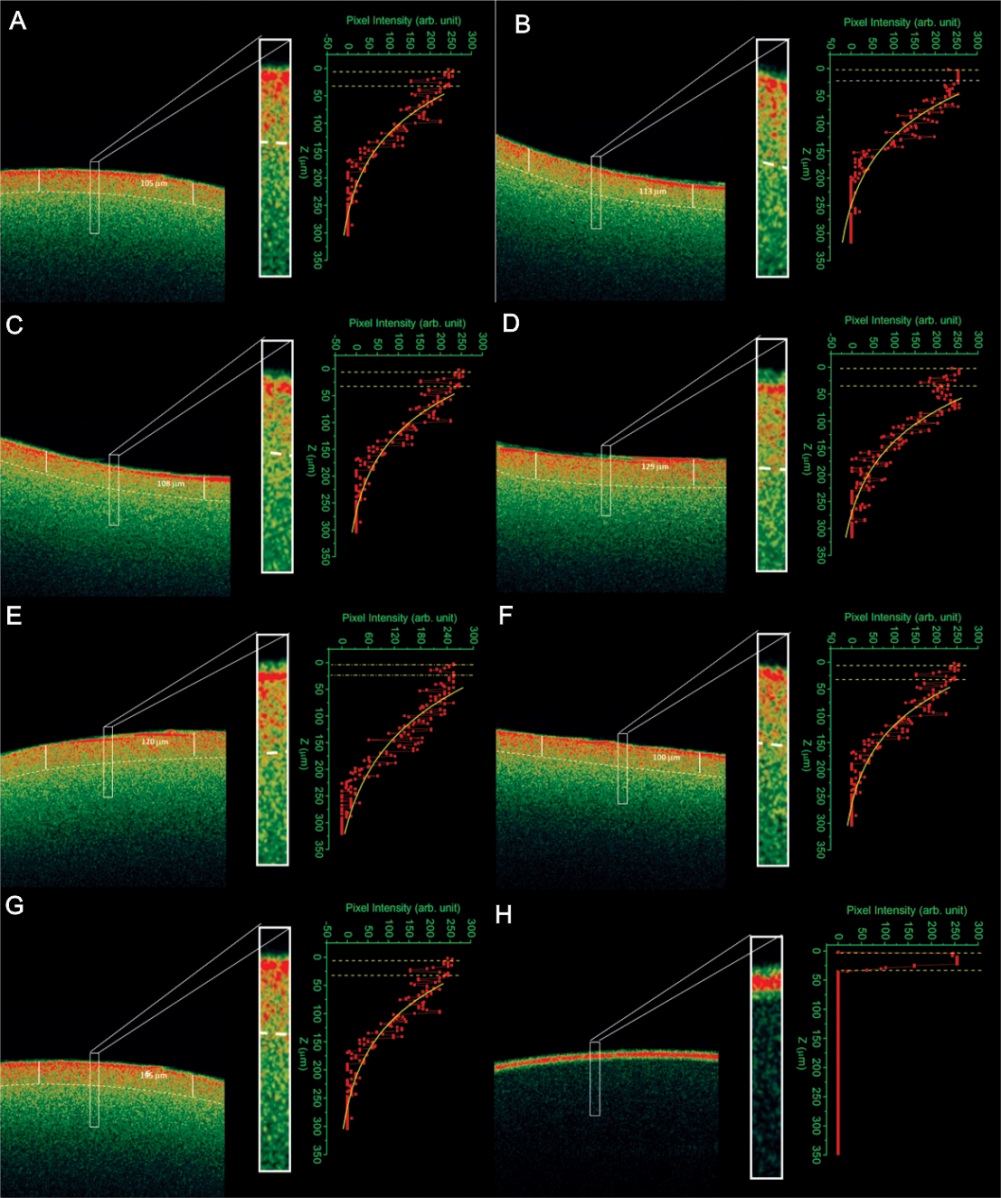
Optical coherence tomography indicating the the extension of changes in the sample structure due to the presence of emergent yeast in catheter discs: (A) *Candida guilliermondii*, (B) *C*. *pelliculosa*, (C) *C*. *haemulonii*, (D) *C*. *lusitaniae*, (E) *C*. *famata*, (F) *C*. *ciferri* and (G) *C*. *albicans* ATCC 90028. The control (H) is disc free of biofilm.

Samples were analyzed using OCT, which exhibited the extension of changes in the catheters discs. In the OCT images, red shades represent higher scattering areas. Pixel intensity distributions (A-scan) of selected regions are also presented, indicating that the amplitude of the scattered light decrease at deeper areas under the biofilm surface (Fig 2). The OCT results are independently among *Candida* strains and no species-specific pattern.

**Fig 3:**
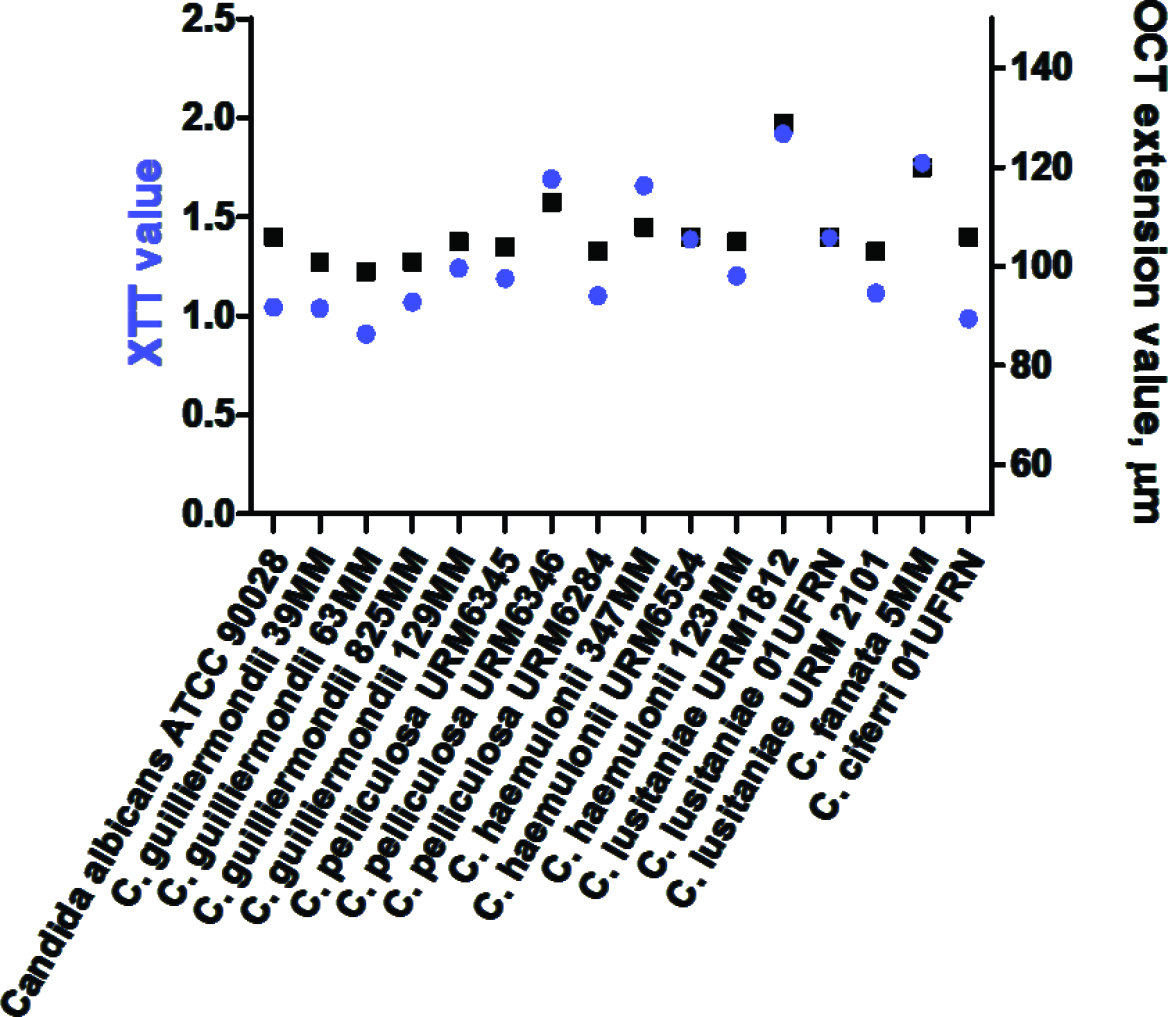
Ample structure changes and metabolism of emergent *Candida* biofilms observed by optical coherence tomography (OCT) and oxidative activity (XTT). The results correlation shows positive distribution (Pearson correlation test, r = 0.8752, *p* < 0.0001).

**Fig 4:**
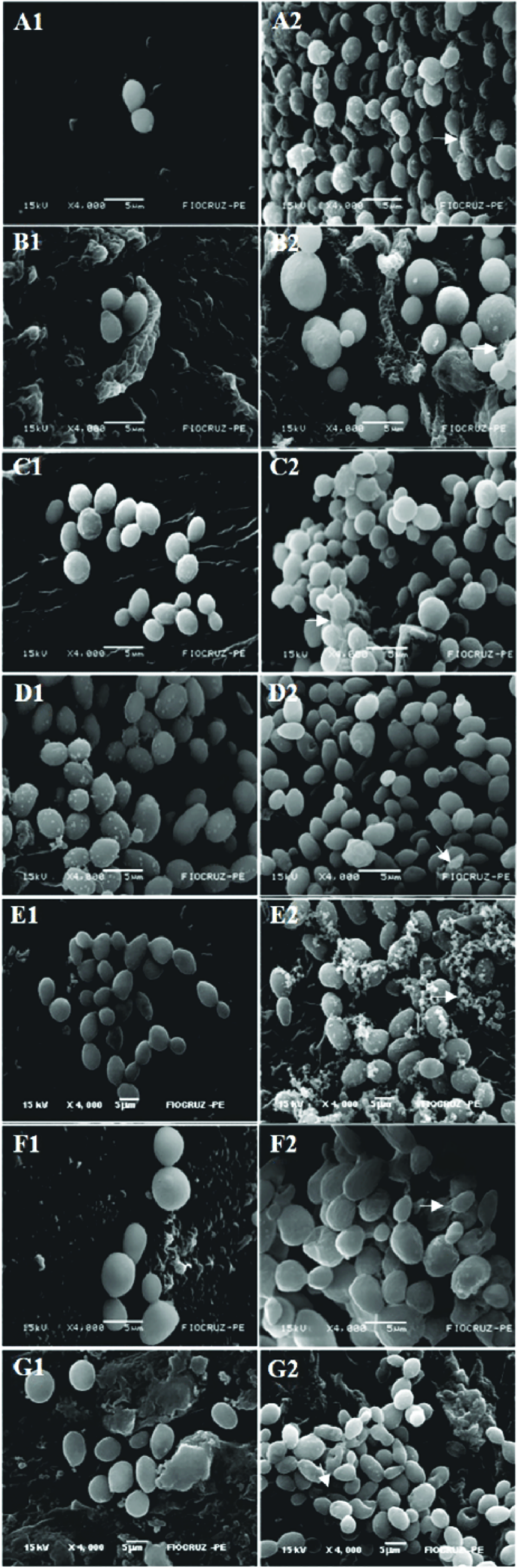
Scanning electron microscopy of 24h (A1-G1) and 48h (A2-G2) biofilms formed on catheter discs for emergent *Candida* strains. *Candida guilliermondii* (A1, A2), *C*. *pelliculosa* (B1, B2), *C*. *haemulonii* (C1, C2), *C*. *lusitaniae* (D1, D2), *C*. *famata*, (E1, E2) and *C*. *ciferri* (F1, F2). The control (G1, G2) is *C*. *albicans* ATCC90028. Arrow indicates the presence of the extracellular matrix within the biofilm. Magnification x4.000.

The correspondence between the XTT and the measured OCT values are shown in Fig 3. There was a significant positive correlation between oxidative activity and optical coherence tomography in biofilm development (Pearson correlation test, r = 0.8752, *p* < 0.0001). Furthermore, correspondence in results were visually demonstrated by SEM through observation of cells attachment, multilayer and morphologic characteristics (Fig 4).

### Qualitative analyses of biofilms: Evaluation of architecture in catheter discs

During the first 24 hours of incubation, *C*. *lusitaniae* strains exhibited intense biofilm formation, followed by *C*. *famata*, *C*. *haemulonii* and *C*. *albicans* reference strain. However, the other species (*C*. *ciferri*, *C*. *pelliculosa* and *C*. *guilliermondii*) showed sparse yeast cells adhered to catheter discs’ surface, typical of initial stage of the biofilm formation. Nevertheless, in 48 hours of exposure, all yeast strains showed a mature biofilm on the surface of the discs.

## Discussion

The *Candida* species evaluated in this study are rarely described in clinical cases of invasive fungal infections. However, when this condition occurs, these yeasts are commonly isolated from the bloodstream of patients with poor prognostic [16–17].

The fact that these emergent yeast species are able to form biofilms may be clinically relevant. In fact, Tumbarello et al. [18] found that patients with candidemia caused by non-biofilm-forming *Candida* spp. strains had better outcomes. In contrast, the biofilm-forming strains showed a significant association with poor prognoses.

In addition, Iturrieta-Gonzalez et al. [19] described that *Trichosporon*, another emergent yeast genus, showed levels of biofilm formation similar or greater than those described for *Candida* spp. and that biofilm-forming cells were at least 1,000 times more resistant to antifungals than their planktonic counterparts. These data reinforce the likely importance of biofilm formation by the rare *Candida* species used in the present study.

The evaluation using the XTT reduction and the biofilms images demonstrated the metabolic viability inside matrix in all tested yeasts, with significant differences between species and within different strains of the same species. In contrast, *C*. *guilliermondii* isolates showed no significant intra-specific variations, demonstrating a homogenous pattern of viability for sessile cells present in the biofilms. Interestingly, the high XTT OD 492nm obtained for *C*. *lusitaniae* (URM 1812) biofilm is in agreement with the literature because emergent yeasts may form viable multicellular structures in inert surfaces as catheters and invasive devices or colonize valves in humans [12].

Based on light scattering, OCT images showed the extension of changes inside the catheter structure, indicating the pathogenicity of these emerging yeast species. A clear image difference can be observed among the samples with and without (control) biofilm (Fig 2). The presence of the biofilm enhances light scattering of the sample, increasing the brightness of undersurface areas of the OCT biofilm images. Scattering can be attributed to the biofilm microstructure on the catheter. Thus, it was observed that the OCT values are higher for those isolates with the high XTT activity. Few studies have been developed using these techniques to clarify structure and metabolism of *Candida* biofilms [12, 15]. This is the first study to explore OCT technique to evaluate emergent clinical yeasts. The OCT technique offers advantages once it clearly proved to be non-invasive, real-time, privileging in-situ analysis of biofilm layers. Moreover this biofilm keep its original structure without the performance of a destructive process enabling the assessment of biofilm roughness and surface area [20].

Through of the scanning electron microscopy, it was visually verified that all biofilm formation stages occurred in the yeasts tested within 48 hours. Thus, with a significant increase of cell proliferation with amorphous material, representing the extracellular matrix and featuring a mature biofilm. Interestingly, rare yeast cells were adhered to the surface of the catheter disc during the first 24h in *C*. *ciferri*, *C*. *pelliculosa* and *C*. *guilliermondii*. *C*. *lusitaniae* (URM 1812) showed intense cell adhesion at this time of evaluation. This species showed a higher number of yeast cells adhered to the discs and intercellular adhesion. In addition, we observed in scanning electron microscopy that the biofilm of all species was composed of blastoconidia, but did not present filamentous forms. All emergent *Candida* biofilms appeared as discontinuous layers of blastoconidia anchored to the surface and rich in extracellular matrix but not hyphae, which was in accordance with the findings of Silva et al. [21] after evaluating *C*. *glabrata*, *C*. *parapsilosis* and *C*. *tropicalis* biofilm. As demonstrated by Chandra et al. [22] both RPMI as YNB media are used to form well-structured biofilms, however YNB enhances budding reproduction and RPMI stimulates filamentation. Their data indicate that biofilm growth was not morphology specific.

Sparse investigations upon *C*. *albicans* biofilm architecture can be found in the literature [23]. However, currently, there are no studies characterizing the structures of some emergent yeasts. Moreover, we observed that *C*. *ciferrii* developed a classic biofilm, with cell viability, indicating the pathogenic potential of this species. Then, with microscopy, we confirmed that in addition to *C*. *albicans*, which was used in the trials as control species, emerging *Candida* species are also able to form biofilms in their entirety, showing the architecture in all stages. In addition, the chemical nature of the catheter, a material made up of polystyrene, polyurethane or polyvinyl chloride enable biofilm formation [24].

The knowledge of pathogenicity mechanisms of yeast biofilms is crucial for the development of new antifungal therapies and diagnostic strategies.

## Materials and methods

### *Candida* strains

Fifteen *Candida* clinical isolates obtained from the Micoteca URM Culture Collection, Medical Mycology Laboratory (MML) from Federal University of Pernambuco and Laboratory of Medical and Molecular Mycology (UFRN), Federal University of Rio Grande do Norte were analyzed in this study. In addition, *C*. *albicans* ATCC 90028 was used as reference strain. The strain descriptions are summarized in Table 1.

**Table 1:** Descriptions of the strains analyzed in the study obtained in the collection of culture URM, Laboratory of Medical Mycology (MML) of the federal university of pernambuco and laboratory of Medical and Molecular Mycology (UFRN), federal university of Rio Grande do Norte.

Microorganism	Strain number	Substrate of origin
*C*. *ciferri*	02UFRN	Blood
*C*. *famata*	05MML	Blood
*C*. *guilliermondii*	39MML	Blood
*C*. *guilliermondii*	63MML	Blood
*C*. *guilliermondii*	129MML	Blood
*C*. *guilliermondii*	825MML	Blood
*C*. *haemulonii*	123MML	Blood
*C*. *haemulonii*	347MML	Blood
*C*. *haemulonii*	URM6554	Nail
*C*. *lusitaniae*	01UFRN	Blood
*C*. *lusitaniae*	URM1812	-
*C*. *lusitaniae*	URM2101	-
*C*. *pelliculosa*	URM6345	Blood
*C*. *pelliculosa*	URM6346	Blood
*C*. *pelliculosa*	URM6384	Blood
*C*. *albicans*	ATCC 90028*	-

-: Substrate of origin uninformed.

* Reference strain

### Emergent *Candida* species identification by MALDI-TOF MS

Homogenous inoculum of yeast cells were grown and maintained on Yeast Extract Peptone Dextrose Agar medium (YEPD). Incubations were performed at 20h and strains were grown aerobically at 37°C according to Lima-Neto et al. [25]. In order to avoid changes in the protein expression pattern, the culture conditions and growth time were standardized as described above. One single colony was directly deposited onto a 196- position target plate (Bruker Daltonik GmbH), in duplicate for each strain.

Aliquots of 1μL of 70% formic acid were added and mixed gently with yeasts. When the liquid medium was almost evaporated, the preparation was overlaid with 1μL of saturated matrix solution {75mg/ml of α-cyano-4-hydroxycinnamic acid (CHCA) in ethanol/water/acetonitrile [1:1:1] with 0.03% trifluoroacetic acid (TFA)}. The isolates were deposited per plate in duplicate, and the matrix-sample was crystallized by air-drying at 25°C for 5 minutes.

The equipment used was MALDI TOF Autoflex III Mass Spectrometer (Bruker Daltonics Inc., USA/Germany) composed of a Nd:YAG (*neodymium-doped yttrium aluminium garnet*; Nd:Y3Al5O12) laser of 1064nm, set to a 66% power. The mass range from 2,000 to 20,000 Da was recorded using a linear mode with a delay of 104ns and an acceleration voltage of +20 kV. The resulting peak lists were exported to the software MALDI Biotyper™ 3.1 (Bruker Daltonics, Bremem, Germany) where the final identifications were achieved.

### Quantitative analyses of biofilms

#### Inoculum and biofilm development for oxidative activity

Yeast strains were cultured aerobically at 37°C for 18h on Sabouraud Dextrose Agar (SDA) and then inoculated in Yeast Nitrogen Base (YNB) broth (Difco Laboratories, Detroit, MI, USA) supplemented with 50mM glucose. After 18h of incubation, cells were harvested, washed twice with PBS (pH 7.2) and resuspended in YNB supplemented with 100mM glucose. *Candida* strains suspensions were prepared to a concentration of 107 cells/mL evaluated using a spectrophotometer Genesis 10S UV-Vis (Thermo Scientific) at 530nm, corresponding to 80% transmittance.

*Candida* species biofilm formation was performed as described by Silva et al. [13]. Briefly, biofilms were grown in commercially available pre-sterilized, polystyrene, flat-bottomed 96-well microtiter plates (TPP; Trasadingen, Switzerland). Aliquots of 100μL of standard cell suspensions of yeasts (107 cells/mL) were transferred into each well and incubated for 1.5h (adhesion phase) at 37°C at 75rpm. After the adhesion phase, cell suspensions were gently aspirated and each well was washed twice with PBS to carefully remove any remaining planktonic cells. In order to allow the growth of biofilm (biofilm phase), 200μL of YNB supplemented with 100mM glucose was added to each well. The plates were incubated for 24 and 48h at 37°C at 75rpm in a shaking TE-424 (Tecnal). After 24h incubation, the medium was aspirated and, biofilms were washed twice with PBS followed by addition of 200μL of YNB medium. All assays were performed in triplicate.

#### Oxidative activity assay

The 2,3—Bis—(2—Methoxy—4—Nitro—5—Sulfophenyl) - 2H - Tetrazolium—5 Carboxanilide (XTT) (Sigma-Aldrich Corp.) was dissolved in PBS at a final concentration of 1mg/mL.

The solution was filter-sterilized and stored frozen at -70°C until use. Menadione solution (0.4mM; Sigma-Aldrich Corp.) was prepared immediately before each assay. For each assay, XTT solution was thawed on ice and mixed with menadione solution at a volume ratio of 20:1. Biofilms were washed twice with 200μL of PBS to remove no adherent cells.

Subsequently, 158μL of PBS with or without glucose at different concentrations, 40μL of XTT and 2μL of menadione were transferred to each well of 96-well plates. The plates were covered with aluminum foil and incubated in the dark at 37°C for 3h. Thereafter, 100μL of the solution was transferred to each well of new 96-well plates. The colorimetric changes were measured at 492nm wavelength using a microtiter plate reader (Spectra-MAX 340; Molecular Devices Ltd., Sunnyvale, CA, USA).

#### Inoculum and biofilm development for optical coherence tomography (OCT) assay

The evaluation of biofilm formation stages was followed using catheter discs. The tests for biofilm formation were developed according Vandenbosch et al. [26]. The strains were grown on Sabouraud dextrose at 37°C for 16h, and then were centrifuged and the supernatant removed for washing the cells with a 0.85% saline solution. Cells were resuspended in 1ml saline solution (Novolab, Geraardsbergen, Belgium) and inocula were subsequently diluted in YNB medium supplemented with 50mM glucose to obtain an optical density of 0.07 at 600nm.

After standardization of fungal inoculum, 1ml of a 1:100 dilution in YNB was added to each well containing catheter discs. The microtiter plates were incubated for 1h at 37°C. Subsequently, the discs were washed (three times) with 1mL saline solution to remove non-adherent cells aseptically transferred to a new well. Subsequently, 1mL of diluted YNB was added with a final glucose concentration of 0.2mM, and plates were incubated for 24h and 48h at 37°C for further analysis by OCT.

#### Optical coherence tomography imaging analysis

Analyzes were performed using OCT technique based on the evaluation of the light scattered by the object, and provides in-depth information of the structures. A 2D image was obtained by the combination of depth-resolved back-scatter light intensity profiles (A-scans) along the section of interest on the sample. In this work, a commercial spectral optical coherence tomography system (Ganymede from Thorlabs Inc.) was used. The image system exploits a super luminescent diode as light source, emitting infrared light, with wavelength centered at 930nm and spectral width of 100nm. With an A-scan rate of 29kHz, this system can produce 29 frames per second with 512 lines per frame and an axial resolution of 5 micrometers. A theoretical model based on an exponential decay fitting is used to describe light intensity distribution (A-scan) in the sample. The light scattering depth of the biofilm (OCT extension value) was determined, and quantified as the inverse of the exponential decay constant of the theoretical fitting.

### Qualitative analyses of biofilms

#### Evaluation of architecture in catheter discs by scanning electron microscopy

The inoculum preparations and biofilm formation were performed as prior described in OCT assays according to Vandenbosch et al. [26]. The biofilms formed on the catheter discs were fixed in 2.5% glutaraldehyde in 0.1M Sodium Cacodylate buffer for two hours. After fixation, the disks were washed with 0.1M Cacodylate buffer three times for 10 minutes for removal of the entire fixative.

Subsequently, post-fixation was performed in a ratio of 1:1 osmium 2% + 0.1M Cacodylate buffer for 30min, allowing a higher contrast of the material.

Two washes were carried out for 10min in 0.1M Cacodylate buffer and distilled water. Subsequently, the samples were submitted to serial dehydration with 30%, 50%, 70%, 90% and 100% acetone for 5 minutes and dried at the critical point. The material was assembled in metal stubs containing carbon tape and silver ink (which serve as an electrons conductor) and then metallized by bombarding with gold, and analyzed using a FEI (Quanta 200 FEG). The processing of the samples followed the proposed protocol Duarte et al. [27].

### Ethics statement

The *Candida* isolates were obtained after review board approval to use the samples from the Micoteca URM Culture Collection, Medical Mycology Laboratory (MML) from Federal University of Pernambuco and Laboratory of Medical and Molecular Mycology (UFRN), Federal University of Rio Grande do Norte. All *Candida* samples were anonymized in accordance to the ethics statement.

### Statistical analysis

Assays were carried out in triplicate for each strain. Statistical analysis was calculated using GraphPad Prism 5 (GraphPad Software, Inc., La Jolla, CA, USA) software. The results from the XTT colorimetric assay were statistically evaluated by analysis of variance with ANOVA and Tukey's test, with significance level *p*<0.05. The presented OCT average values and the corresponding standard deviations were obtained by the evaluation of 75 A-scan of each image. The relationship between quantitative analyses of biofilms was evaluated by the Pearson correlation test (*p*≤0.05).
